# Supplementary material for: The Impact of COVID-19 on High School Student-Athlete Experiences with Physical Activity, Mental Health, and Social Connection
Source: Int J Environ Res Public Health. 2021 Mar 29;18(7):3515. doi: 10.3390/ijerph18073515 (PMC8036482; doi:10.3390/ijerph18073515)
Supplement: Supplementary file 1 [file ijerph-18-03515-s001.pdf]

Supplementary Materials

Supplementary 1
Interview Guide

Interview Guide Development

The interview guide was developed in alignment with the phenomenography methodology. Questions within the guide were developed through an iterative process with experts in the fields of qualitative research (MHM, PDB, KLB, AMB) and with research and clinical experts in fields of physical activity, social connections, and mental health (MHM, PDB, KLB, AMB, DCKS, KOW, HAS). All authors contributed to the development of the interview guide. After an initial development phase, the interview guides were piloted with university and high school students and adjustments made to the questions, as necessary.

Rapport Building Questions

- From your SHRed portal, I see that you participate in X (Y) sports. Can you share a bit about your experience with those/that sport(s)?
- What do you like about playing (or participating in) X (Y) sports?
- What is important to you about XY sport?

Interview Questions

We are interested in understanding your experience with physical activity. By this we mean engaging in sports including your practices, your games, or anything else you do to stay active.

Topic 1: Physical Activity Experience and Changes due to COVID-19

- What's important to you about being physically active?
    - *Prompt:* What does being physically active mean to you, what does it look like, what does it feel like?

Part 1) I want to bring you back to when the restrictions were first put in place and both school and sports were cancelled due to COVID-19,

- If you had to describe it to someone, what was it like for you then?
    - How did you feel?
    - What was important to you at that time?
- Did COVID-19 affect your physical activity at that time when you compare it to before COVID-19?
    - If yes, how?
        - What were you doing?
        - Can you give me an example of what you did before vs. what you did during that time?
        - What was that change like for you?
        - How did it feel?
    - If no, what helped you to stay active during COVID-19?
        - What did you do, or what strategies did you use to remain physically active?
    - Are there any other resources that have helped to support you during this time?

        - What would those resources look like? Who would they come from? How long would they be?

            Interviewer note: Resources can also include those for social connection and mental health

    - Did the changes in your physical activity at that time impact your overall wellness? how so?
        - Please describe that further.
        - Probe: What felt different?
    - How does your social connection impact your overall wellness? How so?
        - Please describe that further.
        - Probe: What felt different?
    - Do you have any other comments or thoughts to help us understand your experience when restrictions first started and school and sports were cancelled?

Part 2) I now want to bring you back to first few weeks where schools started to provide lessons online.

- If you had to describe it to someone, what was it like for you then?
    - How did you feel?
    - What was important to you at that time?
- Did COVID-19 affect your physical activity at that time when you compare it to before COVID-19?
    - If yes, how?
        - What were you doing?
        - Can you give me an example of what you did before vs. during that time?
        - What was that change like for you?
        - How did it feel?
    - If no, what helped you stay active during COVID-19?
        - What did you do, or strategies did you used to remain physically active?
    - Are there any other resources that have helped to support you during this time?
    - What would those resources look like? Who would they come from? How long would they be?
- Did the changes in your physical activity at that time impact your overall wellness? how so?
    - Could you please describe that further?
    - Please describe that further.
    - Probe: What felt different?
- How does your social connection impact your overall wellness? How so?
    - Please describe that further.
    - Probe: What felt different?
- Any other comments or thoughts to help us understand your experience in the first few weeks when schools started online teaching?

Part 3) I now want you to think about how you feel now.

- If you had to describe it to someone, what is it like for you now?
    - How do you feel?
    - What is important to you now?
- Has COVID-19 affected your physical activity now when you compare it to before COVID-19?

- If yes, how?
    - What are you doing?
    - Can you give me an example of what you did before vs. now?
    - What is that change like for you?
    - How did it feel?
  - If no, what has helped you stay active during COVID-19?
    - What are you doing, or what strategies do you used to remain physically active?
  - Are there any other resources that have helped to support you during this time?
  - What would those resources look like? Who would they come from? How long would they be?
- Do you feel changes in your physical activity impact your overall wellness now? how so?
  - Could you please describe that further?
  - Please describe that further.
  - Probe: What felt different?
- How does your social connection impact your overall wellness? How so?
  - Please describe that further.
  - Probe: What felt different?
- Over the last few weeks the government has been lifting restrictions on what Albertans can do, are you aware of any of the changes to the COVID-19 restrictions?
  - If yes:
    - How have you been learning about the lifting of the restrictions?
    - Probe: Where from? What resources?
  - If no:
    - Where would you like to learn about this information?
- Has your engagement in physical activity changed as restrictions have been lifted?
  - If yes, Can you tell me how?
  - Can you give me an example?
- Any other comments or thoughts to help us understand your experience now or since the restrictions began to lift?

Topic 2: Social Support and Physical Activity

We want to know more about your social interactions during COVID-19.

- Tell me about what your ***family*** does to stay physically active
  - Does your ***family*** engage in physical activity together? If yes, how?
  - Is ***family*** support important to you for staying physical active? If yes, how?
  - Can you provide me an example of how your ***family*** has supported you?
- *Interviewer note: Repeat questions replace family with friends, teammates*
- What is your coach doing to help you to stay physically active? Can you describe that further?
- What supports have your coach provided to you to stay physically active?
- Can you provide an example of how your coach has supported you?
- Describe any resources your coach has provided?
- *Interviewer note: Repeat questions replace coach with teacher if in PE or sports performance*
- Is there anyone else who provides you support with your physical activity? If yes, who?

    - *Interviewer note: If yes, repeat questions above*
- Has COVID-19 changed the way you interact with people? If yes, how? If no, how have your interactions remained the same?
    - *Prompt family, friends, teammates, teachers, coaches*

Topic 3: Previous, Current and Potential Helpful Resources
We want know more about resources you have used or would want to have. Resources can include websites, newsletters, zoom webinars, social media posts, or anything that gives you information or support.

- What has helped you to feel connected during COVID-19?
    - What about [*insert item discussed above*] is important to you?
        - *Prompt: feelings, emotions, benefits*
- *Interviewer note: Resources: link to above*
- Are there any resources that have not been helpful?
- I'm going to get you to reflect back in time to when the restrictions due to COVID-19 first happened and schools and sports were cancelled, in your opinion, if we were to have provided resources at THAT time, what information would you have liked to(?) be included in those resources?
    - What would those resources look like? Who would they come from? How long would they be?
- Now reflect back to when schools started going online, what information would be included in those resources?
    - What would those resources look like? Who would they come from? How long would they be?
- Finally, right now as restrictions are being lifted, what would be included in these resources?
    - What would those resources look like? Who would they come from? How long would they be?

Concluding Questions and Remarks
I am now done my formal questions, as I mentioned before, the objective of this study is to learn about how COVID-19 has impacted high school student's experiences with physical activity, and the impact on their mental health. Our ultimate goal is to be able to provide resources to help students better cope during this challenging time and in the future. Are there any other comments you would like to add that could help us with this goal?

Supplementary 2

Researcher Characteristics

The research team was established to include experts and trainees from across a variety of disciplines, including kinesiology, psychology, public health, and knowledge translation. This helped to create a strong foundation to address the multifaceted research aim. The team includes experts in a variety of qualitative methodologies (MHM, PDB, KLB, AMB) and with extensive mentorship experience to support the trainees on the project. The project was supported by the anchor author (AMB) who supported the peer debriefing process for HAS and TE. HAS and TE conducted all interviews and HAS completed all analyses with support from AMB. Their researcher characteristics are discussed below.

Interviewer Characteristics

Interviewer characteristics are outlined in brief as the first two authors conducted all interviews and the first author completed all analyses, with support of interpretation and understanding from the second author for interviews she conducted. The first author (HAS) is a graduate student in kinesiology and mental health clinician. Her experience as a clinician, her history of maintaining an active lifestyle, and her research program, kinesiology, provide a unique lens. She has prior experience conducting qualitative research, but no prior experience with phenomenography. The second author (TE) was a research assistant at the onset of the study, then transitioned to a graduate student in kinesiology. She is a certified athletic therapist and maintains an active lifestyle. She had no prior experience with qualitative research and no formal training or experience in mental health. Reflexive memos and peer debriefing helped to support the bracketing and reflexivity processes throughout the study. The first author and anchor author (qualitative expert) helped to support the second author in developing her qualitative

research skills. The first author received additional mentorship from qualitative experts on the research team.

Peer Debrief and Mentor Characteristics

The anchor author is an assistant professor and certified athletic therapist. She has experience with multiple qualitative methodologies and conducting qualitative studies with high school student-athletes. She also has experience as a clinician working with athletes from a variety of background and experience levels.
